# Abraxane, the Nanoparticle Formulation of Paclitaxel Can Induce Drug Resistance by Up-Regulation of P-gp

**DOI:** 10.1371/journal.pone.0131429

**Published:** 2015-07-16

**Authors:** Minzhi Zhao, Chunni Lei, Yadong Yang, Xiangli Bu, Huailei Ma, He Gong, Juan Liu, Xiangdong Fang, Zhiyuan Hu, Qiaojun Fang

**Affiliations:** 1 CAS Key Laboratory for Biological Effects of Nanomaterials and Nanosafety, National Center for Nanoscience and Technology, Chinese Academy of Sciences, Beijing, 100190, China; 2 CAS Key Laboratory of Genome Sciences and Information, Beijing Institute of Genomics, Chinese Academy of Sciences, Beijing, 100101, China; 3 Beijing Proteome Research Center, Beijing Institute of Radiation Medicine, Beijing, 102206, China; University of Kentucky, UNITED STATES

## Abstract

P-glycoprotein (P-gp) can actively pump paclitaxel (PTX) out of cells and induces drug resistance. Abraxane, a nanoparticle (NP) formulation of PTX, has multiple clinical advantages over the single molecule form. However, it is still unclear whether Abraxane overcomes the common small molecule drug resistance problem mediated by P-gp. Here we were able to establish an Abraxane-resistant cell line from the lung adenocarcinoma cell line A549. We compared the transcriptome of A549/Abr resistant cell line to that of its parental cell line using RNA-Seq technology. Several pathways were found to be up or down regulated. Specifically, the most significantly up-regulated gene was ABCB1, which translates into P-glycoprotein. We verified the overexpression of P-glycoprotein and confirmed its function by reversing the drug resistance with P-gp inhibitor Verapamil. The results suggest that efflux pathway plays an important role in the Abraxane-resistant cell line we established. However, the relevance of this P-gp mediated Abraxane resistance in tumors of lung cancer patients remains unknown.

## Background

Drug delivery via nanoparticle-based carriers has shown promising pharmacological improvements in cancer therapy [1, 2] Nanoparticle albumin-bound paclitaxel (Abraxane) has been approved by FDA for use in patients with metastatic breast cancer and Non-small-cell lung carcinoma (NSCLC) [3, 4]. Abraxane is a 130 nm albumin-bound particle form of paclitaxel (PTX), which is a member of the taxane family and an important agent in cancer chemotherapy. PTX acts by binding to microtubules and interfering with the mitotic process [5]. The clinical implementation of PTX was limited by its poor water solubility. Abraxane is less toxic and improves the drug effect in tumor through enhanced permeability and retention (EPR) effect [6]. Furthermore, the transcytosis of albumin-bound paclitaxel across the endothelial barrier is facilitated by its binding to the gp60 receptor and caveolar transport. In the tumor interstitial space, albumin-paclitaxel complexes bind to the Secreted Protein Acidic and Rich in Cysteine (SPARC), which is overexpressed in a majority of tumors [7], to achieve enhanced drug targeting and penetration in tumors [8].

The efficacy of chemotherapy of cancer is impeded by drug resistance, either because tumor cells intrinsically resist drug action, or the tumor cells initially respond to therapy, after which there is selection for cells in the population capable of circumventing drug action [9]. A lot is known about acquired resistance by generation of the cell models in the laboratory. These mechanisms include decreased drug uptake into cells, increased drug efflux, activation of detoxifying enzymes (e.g. cytochrome P450), activation of DNA repair system, and inhibition of apoptotic signaling pathways [10]. Increasing drug efflux by overexpression of ATP-binding cassette (ABC) transporters is a common mechanism for cellular resistance to paclitaxel and other anticancer agents such as Doxorubicin (DOX) and vinblastine [10, 11]. ABCB1 belongs to ABC transporter family and encodes a membrane protein P-glycoprotein (P-gp), which is a well-known efflux pump responsible for multiple drug resistance (MDR)[12]. Cells resist PTX were found to exhibit cross-resistance to a variety of other hydrophobic drugs and to have elevated levels of P-gp[10]. Besides the efflux pump, mechanisms of resistance to taxane family drugs also include alterations in the growth characteristics, overproduction of mutant p53 and spontaneous mutations [13, 14], as well as alteration of microtubule composition or dynamics [15], and overexpression of Bcl-2 [16].

It is widely recognized that nano-formulations of drugs can be used to overcome P-gp mediated resistance and a lipid-based PTX nanoparticle was reported to have such feature [17]. In this study, Dong et. al suggested that two major reasons for enhanced cytotoxicity of DOX or PTX lipid-based NPs in P-gp mediated resistance: 1) increased drug uptake by endocytosis that bypasses P-gp and 2) decreased efflux rate through inhibition of P-gp function caused by Brij 78, a surfactant component in NPs [17]. Similarly, the enhanced antitumor activity of Abraxane might relate to increased intra-tumor PTX concentration as reported in one preclinical study [18]. But whether albumin-bound PTX nanoparticle can overcome the drug resistance problem mediated by P-gp is unclear [4]. Zhang et al. show that PTX relapsed tumors developed resistance against paclitaxel, but not to Abraxane [19]. However, whether the mechanism of the resistant tumor in this study is via P-gp is unknown. Stordal et al. reported that resistance of PTX in a cisplatin-resistant ovarian cancer cell line is mediated by P-gp [20]; unfortunately, no test on Abraxane resistance was performed in this work. The excipient of Abraxane is human albumin solution containing albumin, sodium, sodium caprylate and N-acetyl tryptophanate. None of them have been reported to affect P-gp activity. Albumin-bound PTX rapidly decreased its size from 130 nm to proximately 24nm and 10 nm following dilution in plasma to different concentrations. [21]. Gardner et al. developed an assay to quantitate total and unbound paclitaxel in human plasma following Abraxane treatment of patients. They found the unbound form was 6.4% of total drug and does not vary with time [22]. Since PTX is noncovalently bound to Albumin, it is quite possible that PTX might dissociate from albumin and cause the same cytotoxicity and resistance to cells as treatment of free PTX. After Abraxane enters the cell by endocytosis as most nanoparticles [23] or assists by SPARC as mentioned above [8], however, so far there is no detailed study on how Abraxane behaves inside the cell. Presumably, a steady state will be achieved where some drug is bound to albumin and some is dissociated as free form. However, the dissociation constant and time to reach the steady state is not clear. According to the calculation based on the P-gp crystal model, the entrance of the P-gp channel is 2.2 nm and the widest part is 3 nm, which is smaller than many NPs. The size-exclusion effect was assumed to contribute to the anti-resistance effect of some NP-formulated medicines [24]. Thereforeif albumin-bound form is the majority one inside cells, P-gp will not be induced to resist Abraxane. Then, will cells develop resistance against Abraxane? And if yes, what can be the mechanisms involved?

To answer these questions, first we stepwisely increased the concentration of Abraxane added to the drug sensitive non-small-cell lung line (NSCLC) A549. After 6 months we were able to successfully establish an Abraxane-resistant NSCLC cell line (A549/Abr) with an IC50 of 1314.66±25.29nM, whereas the IC50 for A549 sensitive cell is 11.06±1.06nM. We then characterized the changes in cell morphology and cell proliferation. Finally, the transcriptomic profile analysis was carried out on both A549/Abr and A549 using RNA-Seq technology, with the aim to find out the molecular mechanisms involved in the resistance of Abraxane.

## Methods

### Drugs and chemicals

Abraxane was obtained from Celgene Co. (Summit, NJ, USA). PTX, docetaxel (DTX), Doxorubicin (DOX), Cisplatin (CDDP) and fluorouracil (5-FU) were procured from the Zhejiang Hisun Pharmaceutical Factory (China); Verapamil (VP) from Acros Organics Co. (Geel, BE). The antitumor cyclic hexapeptide RA-V [25], isolated from a Chinese natural medicine, the roots of Rubia yunnanensis, was a gift from Ninghua Tan’s group at Kunming Institute of Botany, Chinese Academy of Sciences. Propidium iodide (PI), RNase A and MTT (3-(4,5-dimethyl-thiazol-2-yl)-2,5-diphenyl tetrazolium bromide) were purchased from Sigma Aldrich (St. Louis, MO, USA); DMSO from Amresco Inc. (Solon, OH, USA). Anticancer agents were prepared extemporaneously in complete culture medium immediately prior to *in vitro* use.

### *In situ* TEM

Abraxane was characterized by *in situ* wet-cell transmission electron microscopy (TEM) before the establishment of drug-resistant cell line. Sample was prepared by suspending the Abraxane powder in deionized water, with a final PTX concentration of 10 μM. Sample was added between two silicon nitride window grids in an *in situ* TEM cell. The details of the *in situ* cell structure have been reported earlier [26, 27]. The silicon nitride window grids with 50 nm membrane thickness were purchased from Ted Pella, Inc. Samples were observed with JEOL JEM-2100 TEM system, which was operated under a 200 kV electron acceleration voltage.

### Cell culture and induction of drug resistance

The human non-small-cell lung carcinoma (NSCLC) cell line, A549, was maintained in Dulbecco’s modified Eagle medium/high glucose (DMEM/H; Thermo Scientific, USA) supplemented with 10% (v/v) fetal bovine serum (FBS; Thermo Scientific, USA), 100 U/mL penicillin (Gibco BRL, Grand Island, NY), and 100 μg/mL streptomycin (Gibco BRL, Grand Island, NY) in a highly humidified atmosphere with 5% CO_2_ at 37°C. Cells were passed at preconfluent densities using a solution containing 0.05% trypsin and 0.5 mM EDTA (Life Technologies, Gaithersburg, MD, USA), when the density became 80–90%.

Abraxane-resistant cell line A549/Abr was induced by exposing A549 cells to Abraxane in stepwise increment of concentrations ranging from 10 to 400 nM for over 6 months. To be specific, Abraxane was reconstituted with sterile 0.9% w/v sodium chloride injection (Shijiazhuang No.4 Pharmaceutical Co., China) prior to use, resulting in an effective PTX concentration of 50 μM. When the cells were in logarithmic growth phase, Abraxane was added to the medium to a final concentration of 10 nM as start. From then, at every 24-hour incubation interval, medium was discarded and replaced with fresh one with the same Abraxane concentration. Cells were passed when they were 80% confluent and Abraxane of 10 nM was then added. The concentration of Abraxane was gradually increased by 10–40 nM until 400 nM when the cells had grown steadily in the drug-containing medium. If the cells could not survive, a lower Abraxane concentration was used instead to protect cells from dying. Finally, a cell line resistant to 400 nM Abraxane (named A549/Abr) was derived from A549 after 6 months. For maintenance of Abraxane-resistant cells, the A549/Abr cells were grown in the presence of 100 nM Abraxane. The cells were maintained for at least one week in drug-free medium prior to their use in the experiments. The multiple drug resistance (MDR) characteristics of these A549/Abr cells were identified using various concentrations of anticancer drugs including PTX, DTX, DOX, RA-V, CDDP and 5-FU.

### Cytotoxicity assay in drug sensitive A549 cells and resistant A549/Abr cells

The *in vitro* cytotoxicity of Abraxane, PTX, DTX, DOX, RA-V, CDDP and 5-FU was determined by MTT assay. Briefly, A549 and A549/Abr cells at their logarithmic growth status were seeded in 96-well plates at the density of 4000 cells/well. After 24 h, the medium was aspirated, and replaced with 200 μL of media containing serial dilutions of treatment samples, including Abraxane, PTX, DTX, DOX, RA-V, CDDP and 5-FU. The concentrations of Abraxane and PTX used in each group were 1, 5, 10, 20, 40, 100, 500, 2000 nM. For DTX, the concentrations were 0.1, 1, 4, 10, 40, 100, 400, 1000 ng/mL; for DOX, 1, 10, 40, 100, 400, 1000, 4000 nM; for RA-V, 0.0002, 0.002, 0.02, 0.2, 2, 20, 100 μg/mL; for CDDP, 0.2, 1, 4, 10, 20, 40, 100, 400 μM. Finally, the concentrations of 5-FU were set as follows: 0.01, 0.1, 0.4, 2, 4, 10, 20, 40 μg/mL. After incubation for 48 h at 37°C, the drug-containing growth medium was replaced with 100 μL medium containing 10 μL of MTT (5 mg/mL in PBS). After 4 h, the culture solution was removed, leaving behind the precipitate. Thereafter, 150 μL of DMSO was added to each well to suspend the formazan crystals while vigorously stirring the plates using an automated shaker. The absorbance of each well was read on a microplate reader (Tecan, Switzerland) at a test wavelength of 570 nm. Six wells were used for each drug concentration and the experiment was repeated 3 times. Relative cell viability to untreated control cells was calculated. IC_50_ (concentration resulting in 50% inhibition of cell growth) value for drugs was calculated by SPSS software (version 19.0).

### Cell proliferation assay

We used the MTT method to determine the cell proliferation. Herein, 4×10^3^ cells were plated in 96-wellplates per well. Upon analysis, 100 μL medium containing 10 μL of 5 mg/mL MTT in PBS was added to each well and the cells were incubated for another 4 h at 37°C. The supernatant was then removed, and 150 μL of DMSO was added to each well. The absorbency of each sample was measured with a microplate reader at the wavelength of 570 nm. The surviving cells were measured every day for 7 consecutive days.

### Cell morphology observation

A549 and A549/Abr cells in the phase of logarithm growth were cultured in complete medium with or without 100 nM Abraxane for 24 h. Both cells were examined with the microscope (Leica, Germany) for morphology analysis.

### cDNA Library preparation and sequencing

To prepare samples for RNA-Seq, we cultured A549 and A549/Abr cells in medium with or without 100 nM Abraxane for 4 h. In RNA-seq data analysis, we labeled these four differently treated cells as A549, A549/Abr, A549-100 and A549/Abr-100, respectively. Total RNA was extracted from these cells using TRIzol reagent (Invitrogen) as per the manufacturer’s instructions. RNA concentrations were determined using NanoDrop 2000 (Thermo Scientific). The integrity of RNA samples was determined using 1.2% agarose gel electrophoresis, followed by removal of the residual genomic DNA with RNase-free DNase I (Ambion). Using the Illumina mRNA-Seq library preparation kit, the cDNA library was constructed according to the manufacturer’s instructions. Transcriptome sequencing was then conducted using Illumina Hiseq 2000 Genome Analyzer platform in pair-ended manner with the read length of 100-bp.

### Reads alignment to human genome

TopHat (version 2.0.3) [28] was used to align sequenced reads to the UCSC human reference genome (hg19). We used “bowtie1” parameters during the alignment procedure in TopHat, and no more than two mismatches were allowed for each read.

### Gene expression levels normalization

Aligned results were presented to Cufflinks (version 2.0.2) to calculate the expression level [29]. The gene expression level was measured by the number of fragments per kilobase of transcript per million mapped fragments (FPKM, also known as RPKM [30] in single-ended sequencing experiments). The FPKM method takes account of different gene lengths and sequencing depths between genes and samples, which guarantee the comparison of gene expression among samples.

### Unsupervised Cluster analysis

Logarithmic transformation of gene expression intensity was executed before clustering. Average linkage hierarchical clustering was performed in this study using Pearson distance as the distance measure between genes and samples. Computation and visualization were achieved using heatmap.plus package in R.

### Differentially expressed gene identification

By using Cufflinks and cuffdiff, we identified pairwisely differentially expressed genes (DEG) based on the following criteria: fold change ≥2 (or log2 fold ≥ 1) and FDR ≤ 0.05.

### Gene expression validation by qRT-PCR

Total RNA was extracted as described above and reversely transcribed using the RevertAid First Strand cDNA Synthesis Kit (Fermentas, Glen Burnie, MD). qRT-PCR was performed on the CFX96 Real-Time PCR Detection System (Bio-Rad, Hercules, CA) using Maxima SYBR Green/ROX qPCR Master Mix (Fermentas). Primers were designed using Primer 5 and PrimerBank (http://pga.mgh.harvard.edu/primerbank/index.html). The primer sequences were listed in S1 Table. Transcription levels were normalized to that of glyceraldehyde 3-phosphate dehydrogenase. The samples used for qRT-PCR were the same ones used for RNA-Seq in all groups.

### Gene ontology analysis

WebGestalt [31] was used for Gene Ontology (GO) analysis with the following parameters: “hypergeometric distribution” as statistical method, “BH” as multiple test adjustment, “top10” as the significance level, and “2” as the minimum number of genes for a category. The updated Kyoto Encyclopedia of Genes and Genomes (KEGG) database [32] was used to calculate the enriched pathways of differentially expressed genes.

### Gel electrophoresis and western blotting

To determine the protein level of P-gp, total protein was collected from cultured A549 and A549/Abr cells. Specifically, A549 and A549/Abr cells were washed twice with ice-cold PBS, lysed in RIPA Lysis Buffer (Boster, Wuhan, China) supplemented with 100 μg/mL PMSF. Lysis was incubated with protease inhibitor cocktail (Sigma-Aldrich, St. Louis, MO,USA) for 30 min in an ice bath, then centrifuged at 12,000 rpm for 5 min at 4°C. The concentration of total protein was measured using a BCA Protein Assay Kit (Pierce, USA). Equal amounts of protein extracts (30 μg) were separated on a 10% SDS polyacrylamide gel and then transferred onto a nitrocellulose membrane (Pall Corporation). After blocking at room temperature for 2 h in Tris-buffered saline containing 0.05% Tween 20 (TBS-T) and 5% (w/v) dry powdered milk, the membrane was washed three times for 5 min each with TBS-T. Then the membrane was incubated with primary antibodies to P-gp (Cat. No. 517310; Merck, Darmstadt, Germany) and β-actin (Santa Cruz Biotechnology, CA, USA) overnight at 4°C,washed with TBS-T and then exposed to species-specific horseradish peroxidase (HRP) conjugated secondary antibodies (abcam, Massachusetts, USA) at room temperature for 2 h. Following five washes with TBS-T, the membrane was developed for visualization of proteins by ECL chemiluminescence system (Santa Cruz, USA). A bar chart was made using ImageJ to show the intensities of the protein bands.

### Statistical analysis

Each of the MTT assay, FCM and qRT-PCR was repeated at least three times. And values of one standard deviation (SD) above and below the mean values (mean±SD) were calculated. The significance of difference of the means was tested by the one-way analysis of variance (ANOVA) using SPSS software (version 19.0). Differences were considered significant when P<0.05.

## Results

### Characterization of Abraxane by *in situ* TEM

**Fig 1:**
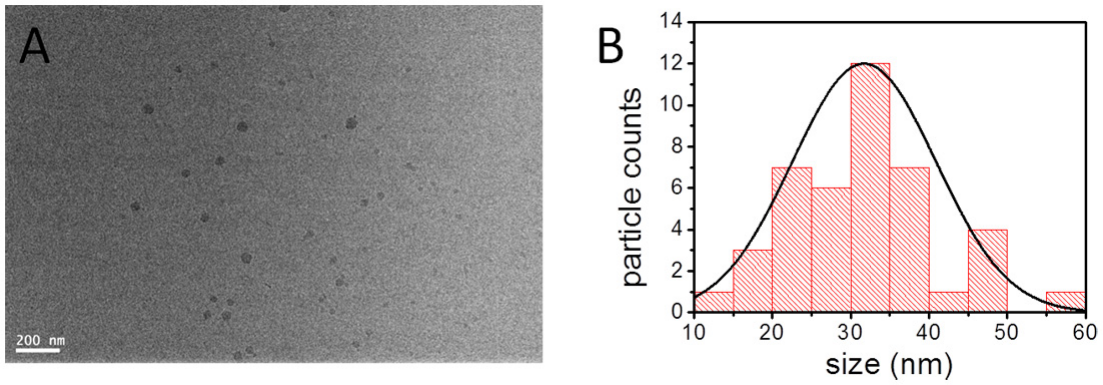
(A) *In situ* liquid cell TEM image of the 10 μM Abraxane solution sample, and (B) the size statistics of the albumin-bond paclitaxel particles obtained from (A). The curve in (B) is the Gaussian fit of the data.

*In situ* transmission electron microscopy (TEM) was used to characterize the albumin-bound Paclitaxel (PTX) nanoparticle (NP) drug in deionized water (see Methods). Specifically, individual particles were suspended in deionized water and were observed to remain stable for at least 3 h (Fig 1A). The particles were roughly round shaped with varied sizes. The sizes of the particles were measured and plotted (Fig 1B). The sizes of the NPs range from 10 nm to 60 nm, with a roughly Gaussian distribution based on more than 40 particles measured. Obtaining the actual Abraxane size in liquid is important for its applications, as the pharmaceutical effect is closely related to the particle size. Our *in situ* TEM measurement is consistent with previous dynamic light scattering measurement [21] in that Abraxane is decomposed to smaller size after suspension.

### A549/Abr cells acquired resistance to Abraxane

**Fig 2:**
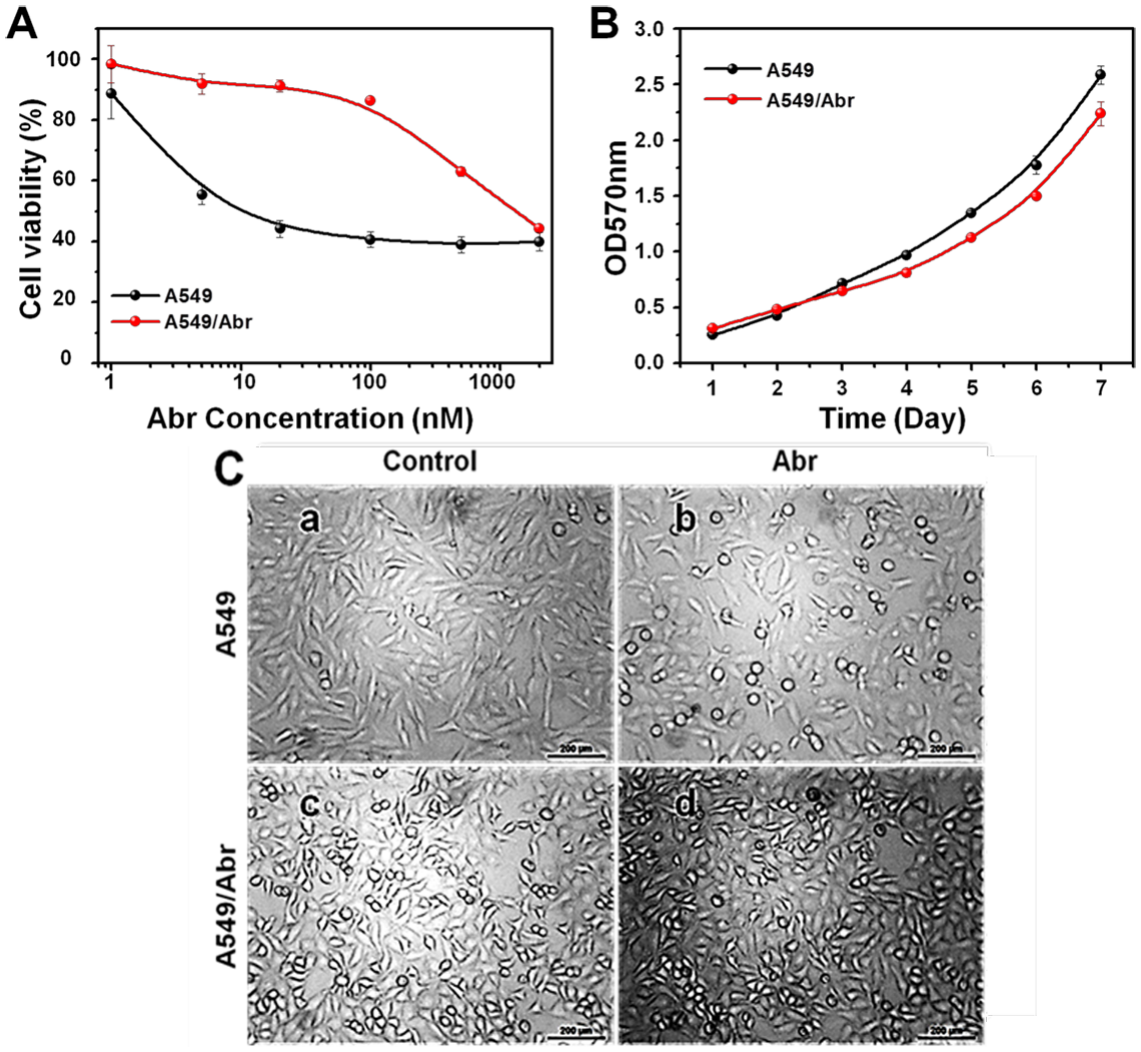
Establishment of Abraxane-resistant NSCLC cell line. (A) The A549/Abr cell line is resistant to Abraxane. A549 (•, black) and A549/Abr (•, red) cells were treated with the indicated increasing concentrations of Abraxane for 48 h. Cell viability was detected using MTT assay. Each point represents the mean±SD of three measurements. IC_50_ is the concentration of Abraxane that inhibits 50% of cell proliferation. The IC_50_ values for the A549 and A549/Abr cells were 11.07 nM and 1314.66 nM, respectively. (B) Growth curves of both cell lines, A549 (black dots) and A549/Abr (red dots). The curves of Abr-resistant cells rise up slowly. The doubling time of A549 cells is 49.56 h and that of A549/Abr cells is 54.65 h. (C) Morphological comparison of A549 cell line and A549/Abr cell line. Effects of Abraxane on the morphologic changes in A549 and A549/Abr cells. Cells were treated with (b and d) or without (a and c) 100 nM Abraxane for 24 h and the morphologic changes were observed by microscopy.

The Abraxane-resistant A549/Abr cell line was established by stepwisely increasing the concentration of Abraxane added to the drug sensitive non-small-cell lung carcinoma (NSCLC) cell line A549 over a period of 6 months as described in Methods. MTT assay showed the IC_50_ values of Abraxane for A549 cells and A549/Abr cells were 11.06±1.06nM and 1314.66±25.29nM, respectively (Fig 2A). The resistance index (RI, ratio between the IC50 of resistant and sensitive cell lines) of the A549/Abr cell line was 118.79, which indicated that an Abraxane-resistant cell line was successfully established. To check whether the resistant cell line acquires multiple drug resistance (MDR) phenotype, we exposed A549/Abr to six other antitumor agents: paclitaxel (PTX), docetaxel (DTX), doxorubicin (DOX), cisplatin (CDDP), cyclic hexapeptide RA-V and fluorouracil (5-FU). IC_50_ from MTT assays and RI values were summarized in Table 1, suggesting that A549/Abr cells exhibited cross-resistance to PTX, DTX, DOX and RA-V, but not to CDDP and 5-FU.

**Table 1:** IC_50_ and RI values of A549 and A549/Abr cells. Effects of selected drugs on both cell lines. RI means resistance index, ratios of IC50_A549/Abr_/IC50_A549_. Data represents mean±SD of at least three separate experiments. The results suggested multidrug-resistant characteristic of A549/Abr.

	IC_50_ (mean±SD)			
Drug	A549	A549/Abr	RI	P
Abraxane(nM)	11.07±1.06	1314.66±25.29	118.79	<0.01
PTX(nM)	8.57±1.49	1412.44±17.91	164.88	<0.01
DTX(nM)	1.10±0.12	297.91±13.65	270.27	<0.01
DOX(nM)	613.21±134.90	1831.07±181.85	2.99	<0.01
RA-V(μg/mL)	0.92±0.17	4.33±0.32	4.71	<0.01
CDDP(μM)	259.67±15.50	250.24±5.63	0.96	>0.05
5-FU(μM)	42.21±3.16	20.74±1.87	0.49	<0.05

### A549/Abr cells show lower proliferation rate than A549 cells

The growth curves of the parent A549 cells and the drug-resistant cells showed that the proliferation rates of A549/Abr cells were lower than that of parental cells (Fig 2B). The population doubling time of A549 cells and A549/Abr cells was 49.56±5.53 h and 54.65±4.87 h, respectively (P<0.05). This indicates that the cell cycle time of A549/Abr is significantly longer than that of A549 cells by 5 hours.

### A549/Abr and A549 cells show different cell morphology with or without Abraxane treatment

We further observed the changes of the cell morphology after treatment with Abraxane in both cell lines. As shown in Fig 2C, before the treatment with Abraxane, A549 cells were healthy and homogeneous in size and shape (Panel a). After treatment with 100 nM Abraxane for 24 h, A549 sensitive cells showed significant morphological changes, e.g., shrinkage in size and becoming more round or oval in shape (Panel b). As for A549/Abr cells (Panel c), more cells had the round shaped morphology, and tended to grow in clusters. Treatment with Abraxane had no obvious effect on the morphology of A549/Abr cells (Panels c and d). This suggests that the resistant cells have acquired significant morphology changes.

### RNA-Seq analyses of A549 and A549/Abr cells with or without treatment of Abraxane

**Fig 3:**
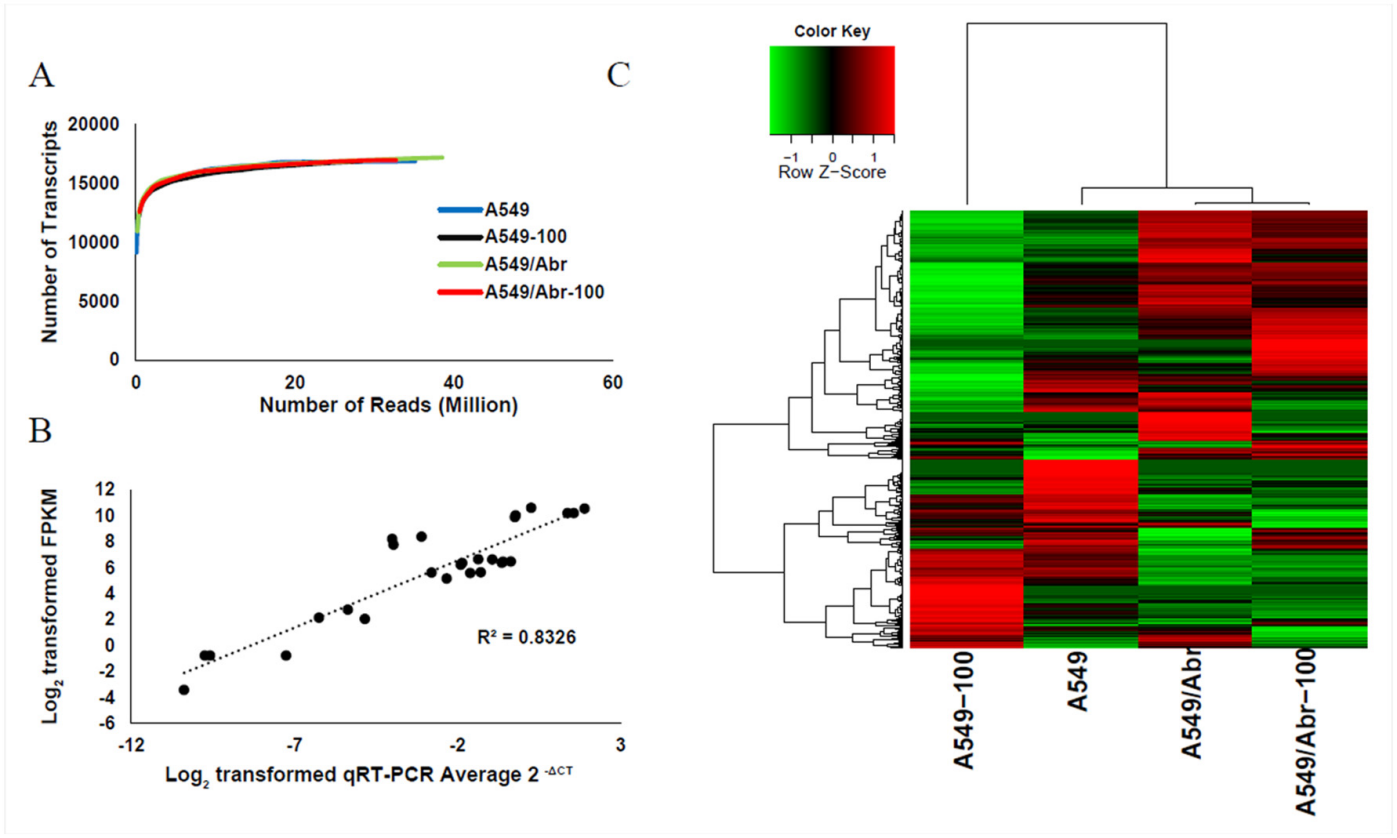
(A) Number of transcripts detected as a function of read depth. X-axis: number of reads (millions) mapped to the genome; Y-axis: the number of transcripts detected in genes from the National Center for Biotechnology Information (NCBI) database. (B) Correlation between qRT-PCR and RNA-Seq for selected genes in A549, A549-100, A549/Abr and A549/Abr-100 cells. The selected genes are ABCB1, ABCC1, ABCG2, MGST1, BCL2, BAX, TP53, ANXA2, HIF1A. (C) Cluster analysis of expressed genes in four cell types. Expression intensities are displayed from green (low expression) to red (high expression).

To understand the mechanisms leading to MDR of A549/Abr cell line, high-throughput sequencing was performed on the Hi-Seq2000 sequencing platform in parallel. We compared the genome-wide expression profile between A549 and A549/Abr cells with (A549-100 and A549/Abr-100) and without treatment (A549 and A549/Abr) of 100 nM Abraxane for 4 h. The quality of the sequencing reads was satisfied for further analysis (S1 Fig). In addition, expression profiles were highly reproducible between the two biological replicates (r^2^ = 0.84, p <0.0001) (S2 Fig) sequenced for A549 cell line. Approximately 21–38 million reads (100bp paired-end) were obtained and mapped to the human genome. Despite the different sequencing depths obtained for these four samples, the number of transcripts detected approached saturation for all of the samples (Fig 3A), similar to a previously reported transcriptome study [33]. The gene expression levels were measured by the number of fragments per kilobase of transcript per million mapped fragments (FPKM) as described in Methods.

### Validation of the next generation sequencing (NGS) data by qRT-PCR

To verify the sequencing result, we quantitated the expression of resistance-related genes in these four cell populations using qRT-PCR. Nine genes including three ABC transporters (ABCB1, ABCC1, ABCG2); the drug metabolism enzyme Glutathione S-Transferase Pi 1 (GSTP1); three apoptosis-related genes: B-cell CLL/lymphoma 2 (BCL2), BCL2-associated X protein (BAX) and tumor protein P53 (TP53); a calcium dependent phospholipid binding protein gene annexin A2 (ANXA2) which was identified as highly expressed in MCF-7/ADR (also called NCI/ADR-RES), a p-glycoprotein overexpressing cell line resistant to adriamycin [34, 35]; and hypoxia inducible factor 1, alpha subunit (HIF-1α) which can promote P-glycoprotein expression [36] were selected. The logarithmic transformations of FPKM values of these genes and their relative copy numbers measured by qRT-PCR were compared using methods described by Yang et. al [33]. Results of this study shown by Fig 3B revealed that RNA-Seq data has a high correlation with qRT-PCR data (p < 0.0001; R^2^ = 0.8326).

### Hierarchical Clustering analysis

Unsupervised hierarchical cluster (Fig 3C) analysis showed that A549/Abr and A549/Abr-100 were clustered together, indicating that they shared similar expression patterns. Furthermore, A549/Abr and A549/Abr-100 clustered with A549 before they clustered with A549-100. The large variation of expression pattern of A549-100 compared to others suggests that the molecular network of sensitive cells has a large perturbation when short-term exposed to drug and the perturbation was much different from the resistant cells which had been long-term exposed to Abraxane.

### Unique classes of differentially expressed genes by the treatment of Abraxane

Using a 2-fold and FDR ≤ 0.05 cutoff for differential expression identification (see Methods), we found 61 differentially expressed genes (DEG) between A549 group and Abraxane treated A549-100 group. Gene Ontology analysis found that these genes were significantly enriched in cytoskeleton, basolateral plasma membrane, and intrinsic apoptotic signaling pathway in response to endoplasmic reticulum stress (Table 2). Genes such as DNA-damage-inducible transcript 3 (DDIT3), cation transport regulator homolog 1 (CHAC1) and tribbles pseudokinase 3 (TRIB3) which are related with apoptosis [37–39], were up-regulated in A549-100 cells, suggesting Abraxane treatment induced apoptosis in A549 cells. Most of the genes related with cytoskeleton were down-regulated in A549-100 compared with A549 cells, which might be the feedback response to the pharmacological effect of Abraxane, as the effective component PTX can stabilize the microtubules by preventing depolymerization thus affecting the cytoskeleton.

**Table 2:** GO enrichment of differentially expressed genes between A549 and A549-100.

GO terms	adjP
cloaca development	0.0155
regulation of epithelial cell differentiation	0.0155
intrinsic apoptotic signaling pathway in response to endoplasmic reticulum stress	0.0155
developmental process	0.0155
photoreceptor cell outer segment organization	0.0155
regulation of developmental process	0.0258
nephric duct morphogenesis	0.0282
system development	0.0282
organic acid biosynthetic process	0.0282
cardiac muscle fiber development	0.0282
structural constituent of cytoskeleton	0.011
basolateral plasma membrane	0.0018
cell-cell junction	0.0021
adherens junction	0.0168
anchoring junction	0.0177
cytoskeleton	0.0323
keratin filament	0.0449
tight junction	0.0449
cell junction	0.0449
occluding junction	0.0449

### P-gp was significantly up-regulated in A549/Abr cells

**Fig 4:**
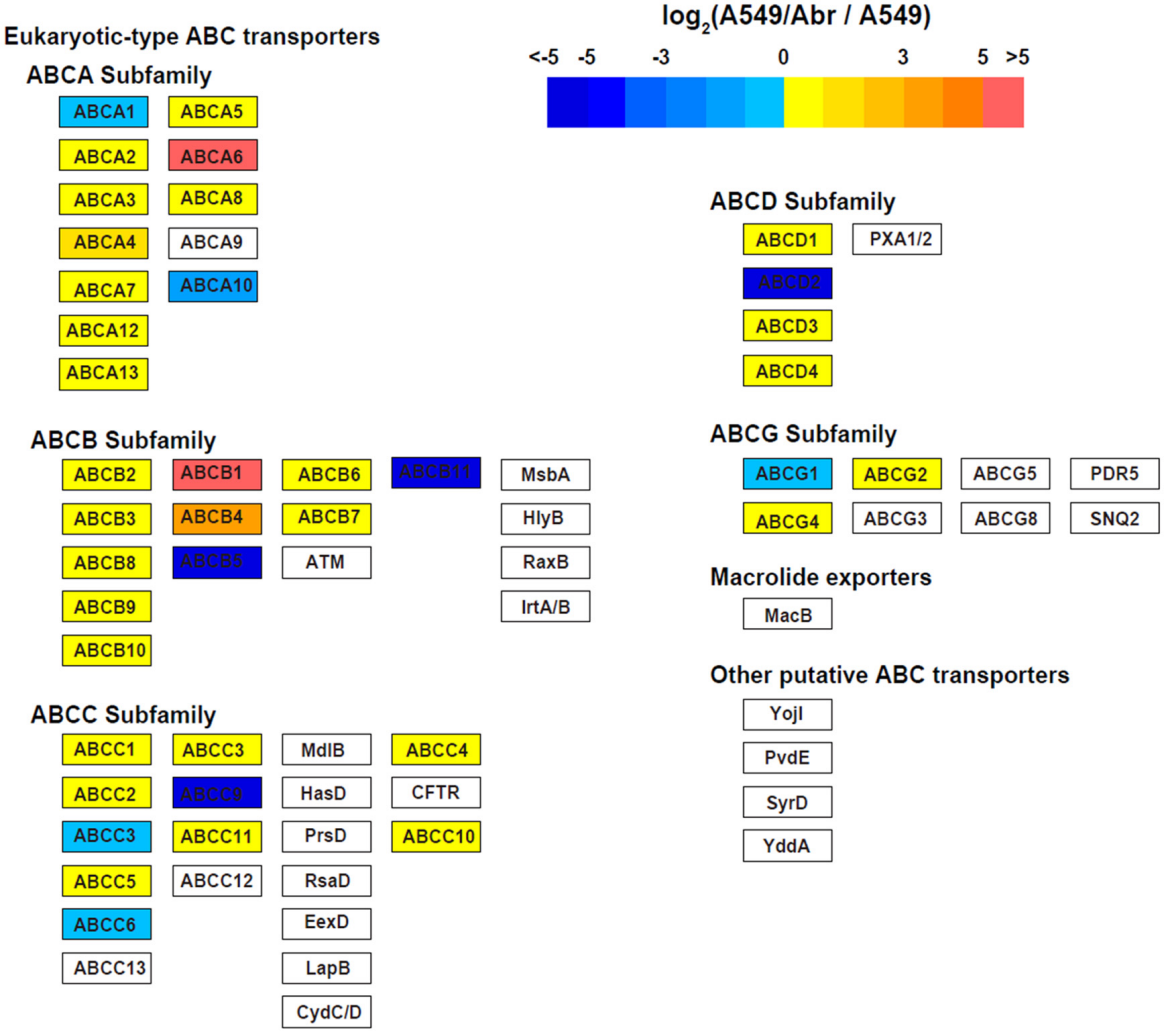
ABC transporters are significantly up-regulated in MDR cells compared with A549 parental cells. This figure shows the expression changes between A549/Abr and A549, other comparisons are included in S3 Fig.

Differential expression analysis using Kyoto Encyclopedia of Genes and Genomes (KEGG) database[32] identified that ABC transporters were up-regulated in MDR cells in A549/Abr *vs* A549, A549/Abr-100 *vs* A549, A549/Abr *vs* A549-100, and A549/Abr-100 *vs* A549-100 comparisons, of which ABCB1 (P-gp) changed most significantly (log_2_(FC)>9) (see Fig 4 and S3 Fig). 35 significantly differentially expressed genes (DEGs) between A549 and A549/Abr were shown in Table 3, among which 16 were up-regulated and 19 were down-regulated. Gene Ontology (GO) enrichment of DEGs between A549 and A549/Abr cells were summarized in S2 Table. Terms such as “response to wounding”, “response to stimulus”, “inflammatory response” and “extracellular region” were identified, indicating genes constitutively expressed in resistant cell lines with response to long-term Abraxane stimulation.

**Table 3:** Differentially expressed genes between A549 and A549/Abr cells.

Gene	A549-P	A549-R-0	log_2_(Fold change)	Up/Down regulation
ABCB1	0.0925563	47.8809	9.0149	↑
CDH11	0.0833912	6.40447	6.26304	↑
DMD	0.0385045	2.78563	6.17683	↑
SLC12A3	0.0718359	1.3755	4.2591	↑
KRT71	0.142844	1.79994	3.65543	↑
PCDH7	0.312025	2.51407	3.01029	↑
UG0898H09	0.154445	1.04428	2.75735	↑
MARCH4	2.73803	14.6769	2.42234	↑
PSG5	0.511795	2.63347	2.36333	↑
MEG3	1.7302	6.1776	1.8361	↑
GPR162	1.09505	3.79453	1.79292	↑
WNT5A	1.22759	3.37723	1.46001	↑
KREMEN2	1.60257	4.33324	1.43506	↑
CAPG	19.9343	52.2711	1.39076	↑
AXL	49.8416	121.681	1.28768	↑
CACNA1G	1.97871	4.00118	1.01587	↑
FGG	7.99229	0.0265285	-8.23492	↓
HSD11B1	12.9429	0.127323	-6.66752	↓
GPC6	2.44818	0.0290896	-6.39506	↓
RPPH1	1591.31	20.0729	-6.30882	↓
CEACAM5	1.62345	0.0215542	-6.23495	↓
RMRP	2097.47	37.0419	-5.82335	↓
FGB	4.09976	0.0747985	-5.77639	↓
LPHN2	0.674788	0.0131037	-5.68639	↓
CEACAM6	41.8537	0.817423	-5.67813	↓
SAA2	114.639	2.39198	-5.58275	↓
SAA1	702.897	17.4412	-5.33274	↓
SLC7A7	6.94533	0.17289	-5.32811	↓
PPP1R14D	6.28935	0.166546	-5.23892	↓
NACA2	419.587	11.4161	-5.19983	↓
MYL9	7.79545	0.233606	-5.06048	↓
MAN1C1	26.4085	0.847124	-4.96229	↓
CA9	74.4067	2.42393	-4.94001	↓
PIP	169.351	5.67844	-4.89838	↓
HOXD11	3.25867	0.110873	-4.87731	↓

**Fig 5:**
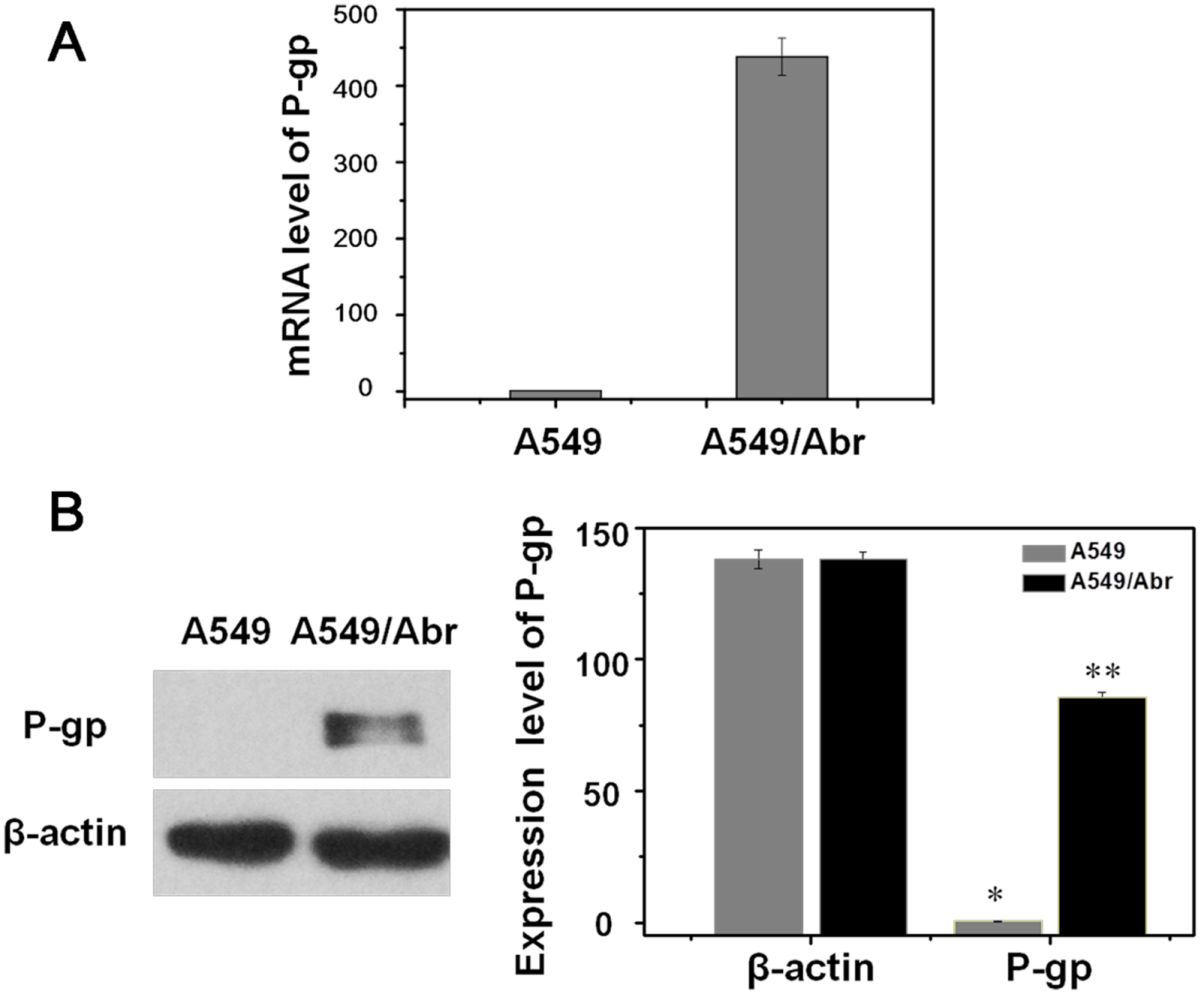
Expression level of P-gp was higher in A549/Abr cells. (A) Real-time PCR analysis of P-gp showed that mRNA level of P-gp increased in A549/Abr cells compared with A549, from 1.0 to 438.01. (B) Western blot analysis of P-gp showed that protein expression level of P-gp increased in A549/Abr cells compared with A549, 85.41 and 0.45 respectively as highlighted with ** versus *. The change is significant (P<0.01) by Student’s t-test.

Since ABCB1 which encodes P-glycoprotein (P-gp) has the most changes, we confirm its change at both RNA and protein expression level in A549 and A549/Abr cells. qRT-PCR was performed to quantitate the mRNA level and it showed that increased in A549/Abr cells compared with A549, from 1.0 to 438.01 (P<0.01, Fig 5A). Western blot analysis was used to determine the relative protein expression of P-gp in A549 and A549/Abr cells. By calculation of the ratio of the integrated intensity of P-gp band to that of the β-actin band in the same sample, we showed that the expression level of P-gp was higher in A549/Abr cells compared with A549 cells, from 0.45 to 85.41 (P<0.01, Fig 5B).

### Verapamil reversed Abraxane resistance in A549/Abr

**Fig 6:**
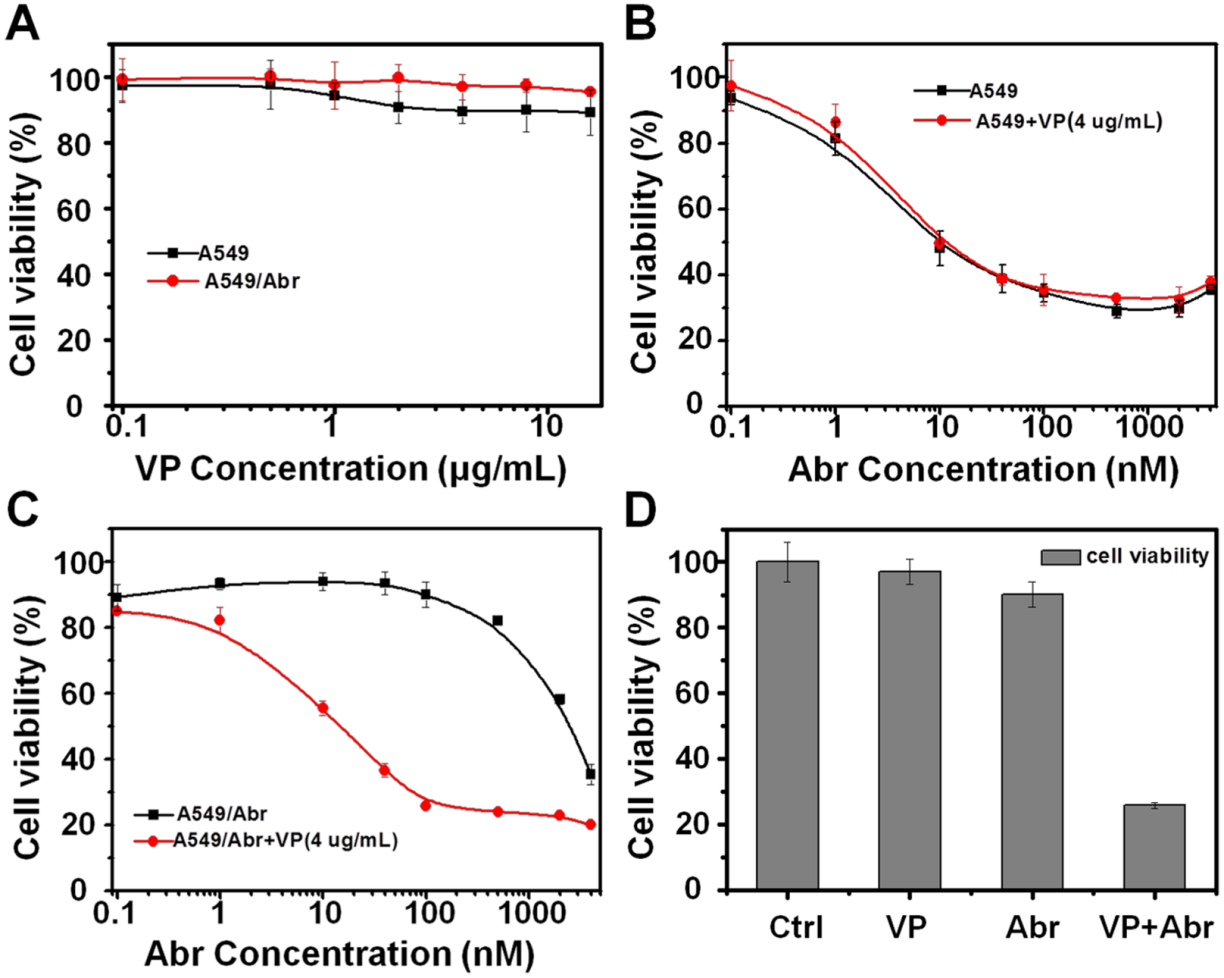
P-gp up-regulation mediates Abraxane resistance in lung cancer cell line. (A) The affection of a P-gp inhibitor verapamil (VP) used alone on A549 and A549/Abr cell viabilities. Both cells were treated with indicated increasing concentrations of VP for 48 h. Cell viability was detected using MTT assay. VP itself didn’t affect much on the viability of A549 and A549/Abr cells. (B-C) Both A549 and A549/Abr cells were treated with indicated increasing concentrations of Abraxane for 48 h at the presence of 4 μg/mL VP. IC_50_ of Abraxane for A549 cells (B) almost remained the same, while that of A549/Abr cells (C) significantly decreased after VP treatment. (D) Comparision of A549/Abr cell viabilities when treated with VP (4 μg/mL) alone, Abraxane (100 nM) alone or 4 μg/mL VP together with 100 nM Abraxane.

To investigate whether P-gp up-regulation is the major cause of A549/Abr cells resistance to Abraxane, P-gp efflux inhibitor Verapamil (VP) was used to see whether inhibition of overexpressed P-gp can reverse Abraxane resistance of A549/Abr cells. Using MTT assay, we evaluated the IC_50_ of Abraxane to A549 and A549/Abr cells with and without treatment of 4 μg/mL VP. As can be seen from Fig 6A, as the control, VP itself didn’t affect the viability of A549 and A549/Abr cells. At the presence of 4 μg/mL VP, the IC_50_ of Abraxane for A549/Abr cells decreased from ~1300 nM to 15 nM (Fig 6C), while that of A549 cells remained 10 nM (Fig 6B). Inhibition of P-gp by VP reversed IC_50_ of Abraxane for A549/Abr to almost the same as its parent cell line. Fig 6D summarized cell viabilities of A549/Abr treated with VP (4 μg/mL) alone, Abraxane (100 nM) alone or VP (4 μg/mL) together with Abraxane (100 nM) for 48 h. These results indicate that when P-gp is inhibited, A549/Abr cell line restore its susceptibility to Abraxane, suggesting that the P-gp up-regulation plays a major role for Abraxane resistance in lung cancer cell line A549/Abr.

## Discussions

Paclitaxel (PTX) is one of the most effective anticancer agents available clinically and it has a wide spectrum of activity against solid tumors [40]. However, its clinical use is impaired by its poor aqueous solubility and even distribution throughout the body, affecting both cancer and normal cells. To overcome these problems, nanocarriers are developed to carry and deliver therapeutics by encapsulating, covalent attaching, or noncovalent binding strategies. In the nanoparticle formulation of PTX, albumin is used as the endogenous carrier of hydrophobic PTX without altering either component or forming covalent bonds. Albumin-bound NP PTX (Abraxane), which was studied in this work, is widely used in clinical treatment of metastatic breast cancer and NSCLC. We aimed to find out the mechanism involved in resistance to nanoparticle formulation of PTX through comparison of Abraxane-resistant A549/Abr cell and its parent A549 cells with NGS technology.

In our study, the RNA-Seq data analysis revealed that the multidrug resistance ABC transporters were up-regulated in MDR cells, among which ABCB1 (MDR1, P-gp) had the most dominant change. Inhibition of P-gp by verapamil (VP) can mostly abolish Abraxane-resistance of A549/Abr cells in vitro (Fig 6). One concern is that verapamil also interacts with calcium channels. However, its function is promoting cancer cell growth by blocking calcium signals that trigger cell apoptosis [41, 42]. This is opposite to the observation that A549/Abr resistance is abolished and cell viability was decreased after treatment of verapamil with Abraxane. Therefore, Abraxane-resistant phenotype is being strongly driven by P-gp overexpression in our newly generated cell line. Considering that PTX has been proved to be a substrate of P-gp [11, 43, 44], and once injected into the body, Abraxane quickly decomposed into smaller albumin-paclitaxel complexes (as shown in Fig 1) and enters tumor cells through association with tumor derived SPARC protein [8, 45]. The observation that overexpression of P-gp plays major role in cell line resistance to Abraxane, same as the common mechanism for free drug, suggests that PTX, the active component in Abraxane, is responsible for the development of drug resistance. Moreover, albumin is over 65 KD, with its physical dimension exceeding the size of ABC transporter’s channel, suggests that PTX is likely dissociated with albumin inside the cell and pumped out by P-gp as a free molecule.

Our study provides for the first time an evidence of Abraxane cellular resistance via overexpression of P-gp in vitro. However, unlike tissue culture, cells in solid tumors are heterogeneous and in a much more complex microenvironment. Delivery of a therapeutic agent has to pass blood vascular system, cross vessel walls and penetrate multilayer of tissue [46, 47]. Therefore, causes of drug resistance in tumors are complicated and cannot be simply explained by mechanisms developed from cell line study. Although there is no report of Abraxane treatment induced resistance in cancer patients so far, we hope that our study will add the understanding of the effect of Abraxane in vitro and in chemotherapy treatment.

Applying the NGS technology that has been demonstrated to provide higher-quality and more informative data than microarray and EST methods [48, 49], we were able to discover the MDR mechanisms through DEG analysis of A549 sensitive and resistant cells. Although in this report, we mainly focused on P-gp which plays a major role in MDR of A549/Abr cells, there might be other mechanisms to explain the higher RI values to taxane family than to other anti-cancer agents as shown in Table 1. In addition, besides ABCB1, many genes such as CDH11, DMD, KRT71, PCDH7, WNT5A and KREMEN2 were up-regulated. CDH11 encodes a cell adhesion protein that is reported to be much regulated in a taxane (docetaxel) resistant cell line [50]. DMD is a large, rod-like cytoskeletal protein. KRT71 belongs to keratin family proteins which are intermediate filament proteins responsible for the structural integrity of cells. And the gene product of PCDH7 is an integral membrane protein that is thought to function in cell-cell recognition and adhesion. WNT5A and KREMEN2 encode proteins associated with Wnt signaling pathway. These suggest that drug resistance against Abraxane might also relate to the enhancement of cell adhesion and the activation of specific signaling pathways. In fact, cell adhesion has been demonstrated to be a mechanism mediated drug resistance [51–53]. Moreover, Hung et al. reported that WNT5A regulates ABCB1 expression in MDR cells through activation of PKA/β-catenin pathway [54]. All these need to be confirmed at the protein level. A proteomic analysis on these cells for protein expression patterns will be performed to understand more resistance mechanisms.

## Conclusions

We successfully established a MDR cell line A549/Abr by exposing A549 cells to Abraxane in stepwise increments of drug concentrations. Through RNA-Seq data analysis and biological validation, our results showed the fact that MDR caused by nanoparticles, Abraxane, is closely associated with ABC transporters, especially P-gp. We speculate that this cell line can provide a tool to further investigate drug resistance mechanisms and facilitate NP-formulated medicine.

## Supporting Information

S1 Fig Reads quality of RNA-Seq in A549 (A), A549-100 (B), A549/Abr (C) and A549/Abr-100 (D) cells. The sequencing reads quality was good enough for later analysis.
(DOCX)

S2 Fig The correlation of two replicates in A549 cells. The expression profiles were highly reproducible between these two biological replicates (R2 = 0.84, p<0.0001).
(DOCX)

S3 Fig KEGG pathway about ABC transporters is significantly upregulated in A549/Abr-100 / A549 (A), A549/Abr / A549-100 (B) and A549/Abr-100 / A549-100 (C). (DOCX)

S1 Table The primer sequences used in qRT-PCR. (DOCX)

S2 Table Gene Ontoloty (GO) enrichment of differentially expressed genes between A549 and A549/Abr cells. (DOCX)
