# Supplementary figures and images for: Abraxane, the Nanoparticle Formulation of Paclitaxel Can Induce Drug Resistance by Up-Regulation of P-gp
Source: PLoS One. 2015 Jul 16;10(7):e0131429. doi: 10.1371/journal.pone.0131429 (PMC4504487; doi:10.1371/journal.pone.0131429)

**S1 Fig**


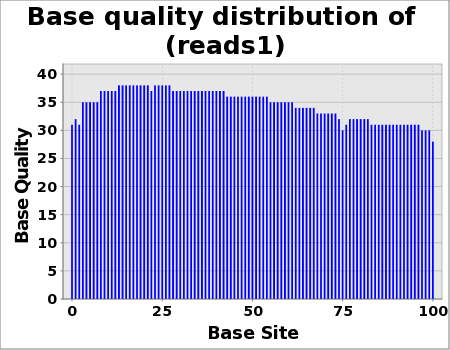

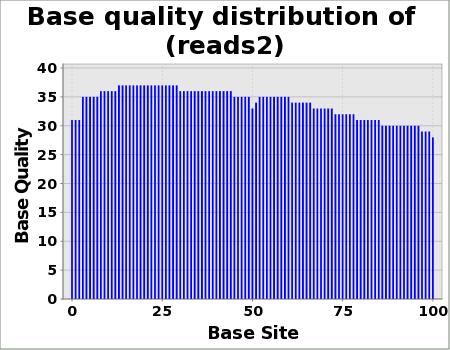


**A** A549 (replicate 1)


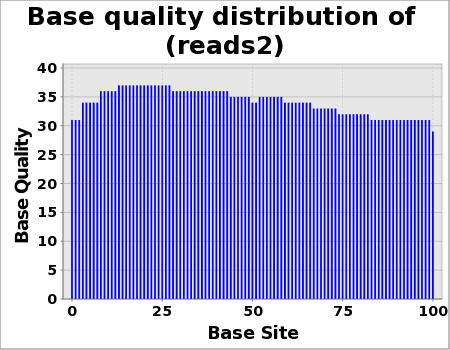

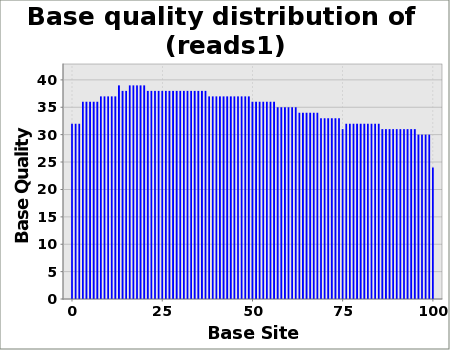


A549 (replicate 2)

**B** A549-100


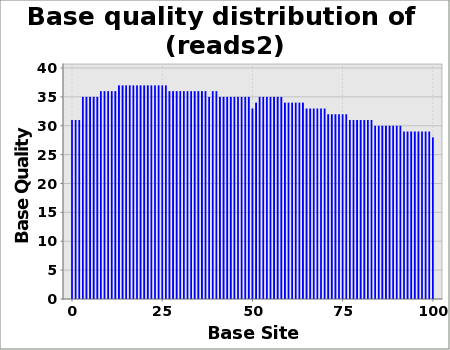

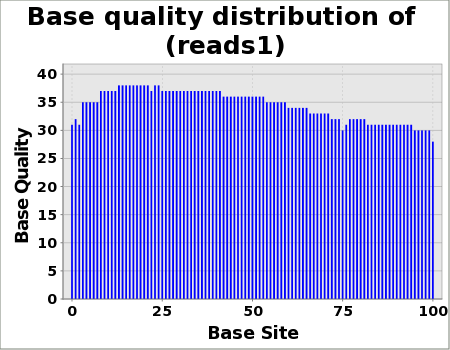


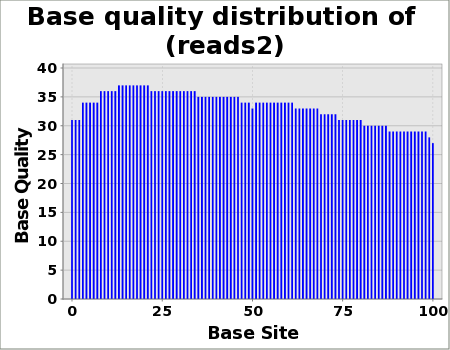

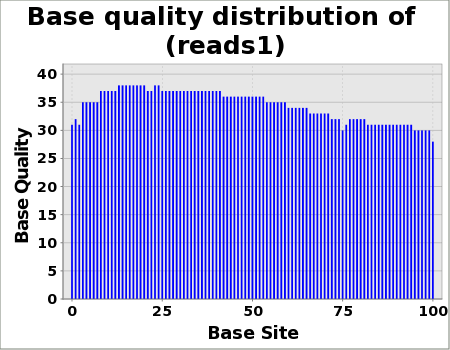


**C** A549/Abr


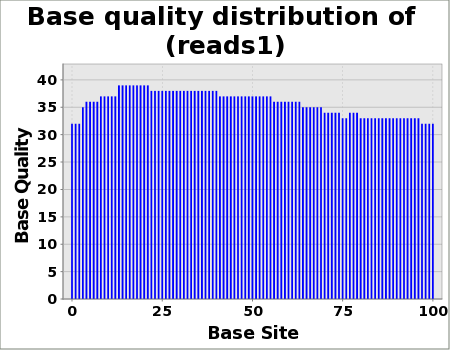

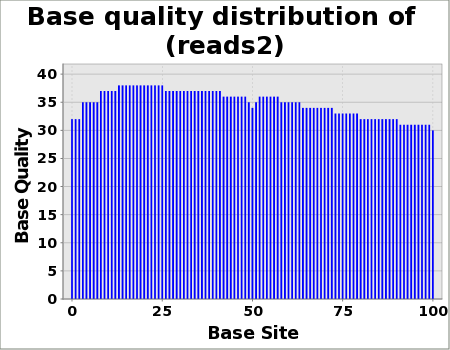


**D** A549/Abr-100

Supplement: S1 Fig — The sequencing reads quality was good enough for later analysis. (DOCX) [file pone.0131429.s001.docx]

**S2 Fig**


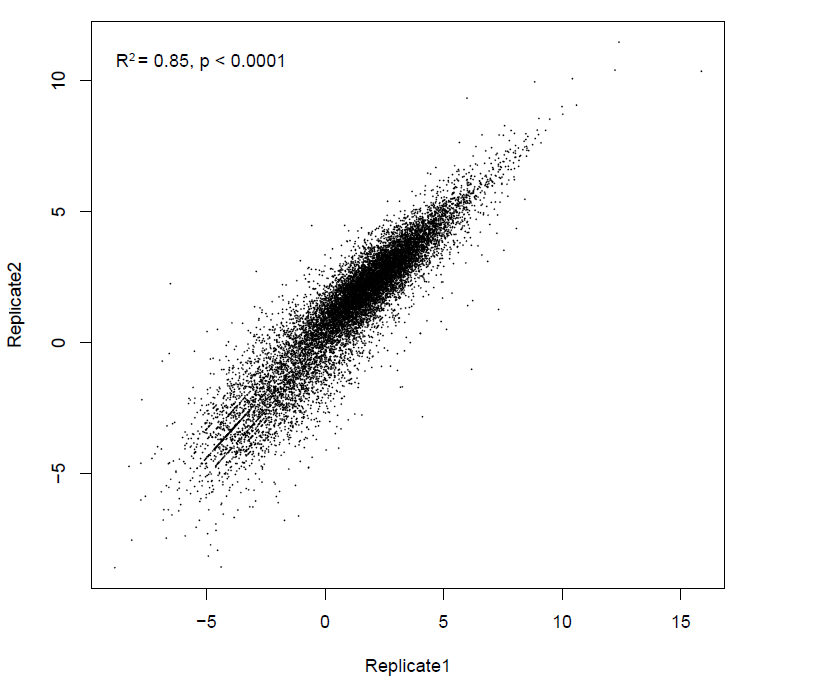

Supplement: S2 Fig — The expression profiles were highly reproducible between these two biological replicates (R2 = 0.84, p<0.0001). (DOCX) [file pone.0131429.s002.docx]

**S3 Fig**


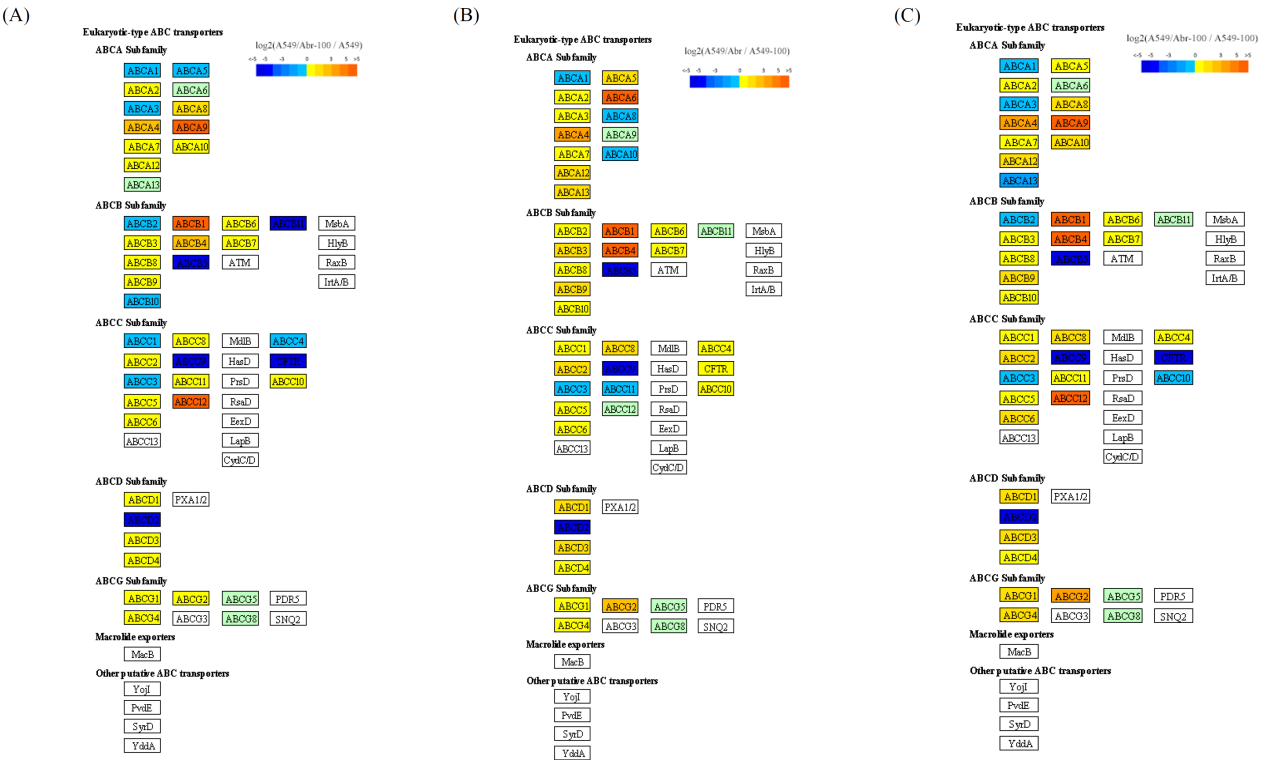

Supplement: S3 Fig — (DOCX) [file pone.0131429.s003.docx]
