# Supplementary material for: Abraxane, the Nanoparticle Formulation of Paclitaxel Can Induce Drug Resistance by Up-Regulation of P-gp
Source: PLoS One. 2015 Jul 16;10(7):e0131429. doi: 10.1371/journal.pone.0131429 (PMC4504487; doi:10.1371/journal.pone.0131429)
Supplement: S1 Table — (DOCX) [file pone.0131429.s004.docx]

**S1 Table.**

| **gene** | **forward** | **reverse** | **fragment** |
| --- | --- | --- | --- |
| ABCB1 | 5'-CCCATCATTGCAATAGCAGG-3' | 5'-TGTTCAAACTTCTGCTCCTGA-3' | 158bp |
| ABCC1 | 5'-CTTGGCCACGTACATTAACATGAT-3' | 5'-CCGATTGTCTTTGCTCTTCATG-3' | 196bp |
| ABCG2 | 5'-GGATTGAAGCCAAAGGCAGAT-3' | 5'-TGACAGCCAAGATGCAATGG-3' | 197bp |
| MGST1 | 5'-CATGCTGCTGGCAGATCA G-3' | 5'-CATTCATCATGTCCACCAGG-3' | 223bp |
| BCL2 | 5'-AGGATTGTGGCCTTCTTTGAG-3' | 5'-GAGACAGCCAGGAGAAATCAAA-3' | 214bp |
| BAX | 5'-CAAACTGGTGCTCAAGGCC-3' | 5'-GCACTCCCGCCACAAAGAT-3' | 188bp |
| TP53 | 5'-CCAGCCAAAGAAGAAACCAC-3' | 5'-TATGGCGGGAGGTAGACTGA-3' | 194bp |
| ANXA2 | 5'-GGTCTCCCGCAGTGAAGTGGACAT-3' | 5'-GGCCAGGCAATGCTTAGGCAACTA-3' | 306bp |
| HIF1A | 5'-GCAAGACTTTCCTCAGTCGACACA-3' | 5'-GCATCCTGTACTGTCCTGTGGTGA-3' | 207bp |
