# Supplementary material for: Abraxane, the Nanoparticle Formulation of Paclitaxel Can Induce Drug Resistance by Up-Regulation of P-gp
Source: PLoS One. 2015 Jul 16;10(7):e0131429. doi: 10.1371/journal.pone.0131429 (PMC4504487; doi:10.1371/journal.pone.0131429)
Supplement: S2 Table — (DOCX) [file pone.0131429.s005.docx]

**S2 Table.**

| GO terms | adjP |
| --- | --- |
| response to wounding | 0.0007 |
| regulation of cellular localization | 0.0008 |
| response to chemical stimulus | 0.0008 |
| response to stimulus | 0.0009 |
| response to lipid | 0.0011 |
| acute inflammatory response | 0.0011 |
| regulation of establishment of protein localization | 0.0011 |
| regulation of cartilage development | 0.0014 |
| regulation of protein localization | 0.0014 |
| inflammatory response | 0.0014 |
| receptor binding | 0.0001 |
| extracellular space | 3.08E-06 |
| fibrinogen complex | 5.34E-06 |
| extracellular region | 1.24E-05 |
| extracellular region part | 1.24E-05 |
| plasma membrane part | 0.001 |
| high-density lipoprotein particle | 0.0089 |
| female genitalia development | 0.0076 |
| vagina development | 0.0076 |
| positive regulation of cytokine-mediated signaling pathway | 0.0076 |
| positive regulation of response to cytokine stimulus | 0.0076 |
